# Cognitive control during audiovisual working memory engages frontotemporal theta-band interactions

**DOI:** 10.1038/s41598-017-12511-3

**Published:** 2017-10-03

**Authors:** Jonathan Daume, Sebastian Graetz, Thomas Gruber, Andreas K. Engel, Uwe Friese

**Affiliations:** 1 Department of Neurophysiology and Pathophysiology, University Medical Center Hamburg-Eppendorf, D-20246 Hamburg, Germany; 2 Institute of Psychology, University of Osnabrück, D-49069 Osnabrück, Germany; 3 Institute of Cognitive Science, University of Osnabrück, D-49090 Osnabrück, Germany

## Abstract

Working memory (WM) maintenance of sensory information has been associated with enhanced cross-frequency coupling between the phase of low frequencies and the amplitude of high frequencies, particularly in medial temporal lobe (MTL) regions. It has been suggested that these WM maintenance processes are controlled by areas of the prefrontal cortex (PFC) via frontotemporal phase synchronisation in low frequency bands. Here, we investigated whether enhanced cognitive control during audiovisual WM as compared to visual WM alone is associated with increased low-frequency phase synchronisation between sensory areas maintaining WM content and areas from PFC. Using magnetoencephalography, we recorded neural oscillatory activity from healthy human participants engaged in an audiovisual delayed-match-to-sample task. We observed that regions from MTL, which showed enhanced theta-beta phase-amplitude coupling (PAC) during the WM delay window, exhibited stronger phase synchronisation within the theta-band (4–7 Hz) to areas from lateral PFC during audiovisual WM as compared to visual WM alone. Moreover, MTL areas also showed enhanced phase synchronisation to temporooccipital areas in the beta-band (20–32 Hz). Our results provide further evidence that a combination of long-range phase synchronisation and local PAC might constitute a mechanism for neuronal communication between distant brain regions and across frequencies during WM maintenance.

## Introduction

Working memory (WM) is the cognitive capacity to maintain and manipulate information for a limited period of time in absence of a continuous sensory stimulation from the outside world^1^. While the organisational structure of the WM system is still under debate, a broad consensus exists about the assumption that successful WM maintenance of stored information requires constant control through the attentional system^2,3^. Other aspects, such as to which extent (if any) WM resources are distributed across the different sensory modalities, remain elusive.

Neurophysiological recordings have strongly contributed to a better understanding of the network dynamics underlying WM. Oscillatory activity related to cognitive control processes were mainly linked to activity found in prefrontal cortex (PFC)^4^. Neural communication between distant brain areas, e.g., during frontal top-down control over posterior areas, has been suggested to be established through phase synchronisation of neural oscillations^5,6^. In fact, phase synchronisation between frontal and posterior areas has been observed particularly at lower frequencies in the delta- (2–4 Hz), theta- (4–8 Hz), and alpha-band (8–12 Hz) during WM maintenance^7–10^ as well as during other cognitive processes^11–13^. Stimulus processing and WM storage activity, on the other hand, may be associated with high-frequency activity in the beta- (13–40 Hz) and gamma-band (>40 Hz)^14,15^, notably in sensory areas^16,17^.

In recent years, the investigation of interactions between different frequency bands has also become a promising target in WM research. In particular, cross-frequency phase-amplitude coupling (PAC), where the amplitudes of high frequencies depend on the phases of low frequencies, is thought to serve as a neural correlate of WM maintenance^18–21^. It has been suggested that PAC during WM maintenance might reflect the interaction between large-scale top-down and local bottom-up processes^22,23^.

Recently, we provided evidence for a potential interplay of cognitive control and visual WM maintenance processes by demonstrating a co-occurrence of frontotemporal theta/alpha phase synchronisation and local theta/alpha-beta PAC within the inferior temporal cortex (IT)^9^. Such a co-occurrence suggests that WM maintenance of visual stimulus material, presumably reflected by PAC in IT, might be controlled by top-down control signals via frontotemporal phase synchronisation in the theta/alpha-band.

In the present study, we aimed at further investigating these processes in dependence of different levels of cognitive control required for maintaining information in visual WM. While keeping the visual WM demands comparable between the two studies, our goal was to manipulate cognitive control required for visual WM maintenance by including a second, auditory WM task. We hypothesized that this auditory WM task would lead to an increase in cognitive control required to coordinate the processing of multiple sensory information in WM. Moreover, we were interested in examining the allocation of domain-specific WM resources by manipulating the difficulty of the additional auditory WM task. Observing alterations in PAC, frontotemporal phase synchronisation, or both would hence provide insights into the underlying neural processes of WM maintenance and cognitive control.

**Figure 1:**
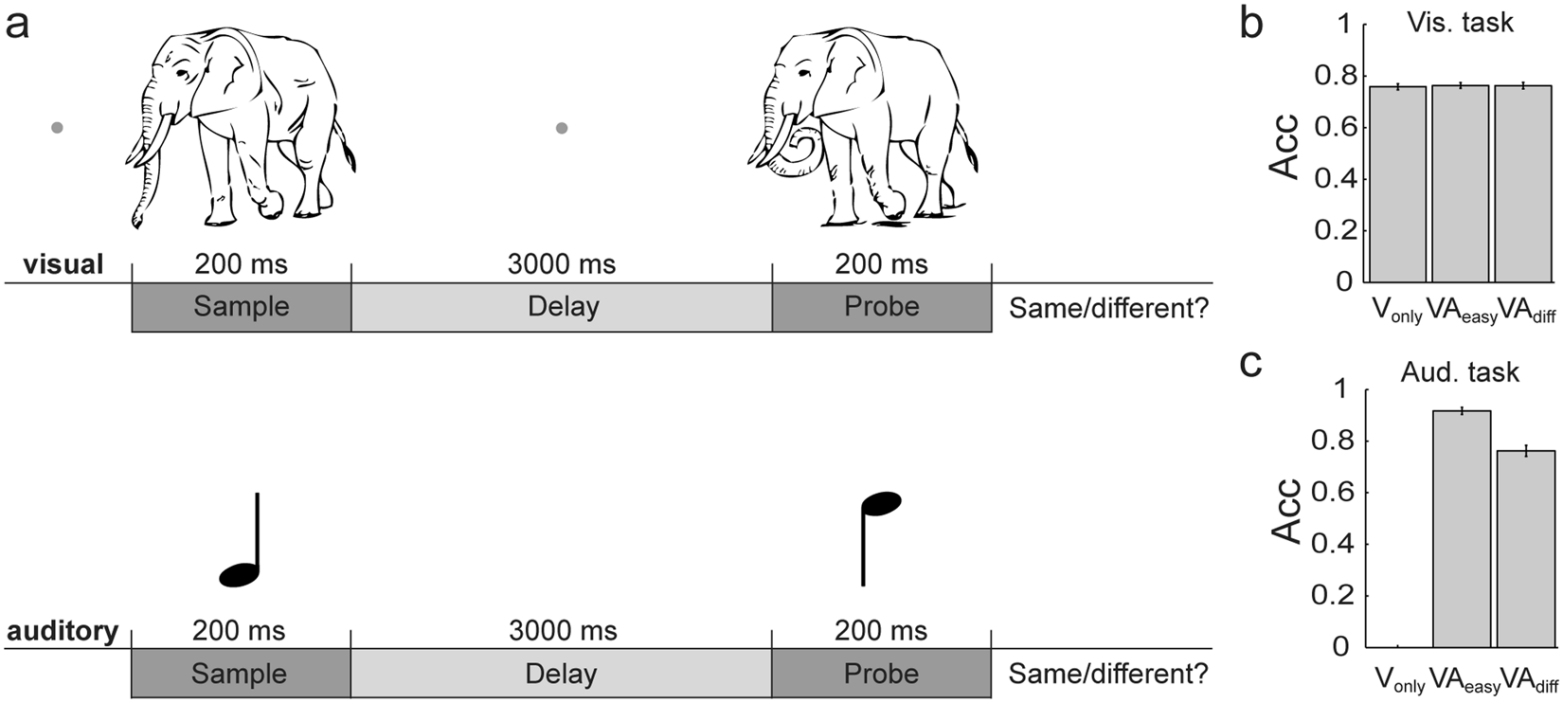
Schematic illustration of the trial design and behavioural results. (**a**) In each trial, participants were simultaneously presented with two consecutive natural visual objects and two sinusoidal sounds delayed by an interval of 3 s. The task was to judge whether probe and sample stimuli were the same or different. In one condition, participants were required to only memorise the visual object and to ignore the auditory one (V_only_). In the other two conditions, both stimuli had to be remembered and compared to the sample stimuli in the respective modality, with one condition consisting of an easy version of the auditory WM task (strong pitch difference; VA_easy_), the other consisting of a difficult version (small pitch difference; VA_diff_). (**b**) Accuracies for the visual WM task in the three conditions. No differences in accuracies were found between the conditions. Error bars represent standard errors of the mean (SEM). (**c**) Accuracies for the auditory task. As expected, participants performed better in the easy auditory task compared to the difficult version. Error bars represent SEM.

We used magnetoencephalography (MEG) to record neural oscillatory activity from healthy participants performing a visual-auditory delayed-match-to-sample task, in which visual and auditory stimuli were always presented concurrently (Fig. 1a). We administered three experimental conditions with different task requirements. In one condition, participants were asked to judge whether the visual probe stimulus matched the visual sample stimulus, while the auditory stimuli were not task-relevant (single-task visual-only, V_only_). In the other two conditions, both, the simultaneously presented visual and auditory sample stimuli, had to be remembered and to be compared to the probe stimuli (dual-task, VA), with one condition encompassing an easy (visual + auditory_easy_, VA_easy_), the other a difficult version of the auditory WM task (visual + auditory_difficult_, VA_diff_).

For the analysis of oscillatory brain activity, we employed measures of spectral power, PAC as well as phase synchronisation. We focused on three independent contrasts: First, we compared an average across all conditions against a baseline window in order to reveal general neural correlates of ongoing WM maintenance processes. Second, by comparing the dual-task conditions against the single-task, we expected to particularly depict differences in cognitive control processes that presumably would be apparent in a modulation of frontotemporal phase synchronisation. Third, by contrasting the VA_diff_ against the VA_easy_ task, we further expected to observe differences in PAC and long-range phase synchronisation, potentially representing modulated processes of WM maintenance and cognitive control due to shifts of resources within the auditory system.

## Results

### Behavioural results

Data from two participants were discarded due to performance at chance level and technical problems, respectively. In the following, behavioural results are reported as means complemented with standard deviations (SDs). Effect sizes are reported as Cohen’s *d* for *t*-tests and partial *η* ^2^ for analysis of variances (ANOVAs). Behavioural data from the remaining 27 participants revealed mean accuracies of 76.46 ± 6.07% for the visual stimuli in the V_only_, 77.54 ± 5.10% in the VA_easy_, and 77.92 ± 4.51% in the VA_diff_ condition (Fig. 1b). A one-way ANOVA with the factor *Condition* resulted in no significant main effect (*F*_(2,52)_ = 1.69; *p* = 0.20, *η* ^2^_*partial*_ = 0.06). Regarding the auditory stimuli, participants responded correctly in 93.43 ± 5.89% of all trials in the VA_easy_ condition and in 76.80 ± 10.83% of all trials in the VA_diff_ condition (no response to the auditory stimuli was required in the V_only_ condition; Fig. 1c). A paired and two-sided *t*-test revealed significantly lower accuracies for the VA_diff_ as compared to the VA_easy_ condition (*t*_(26)_ = −11.78; *p* < 0.01; *d* = −2.27).

### Spectral Power

**Figure 2:**
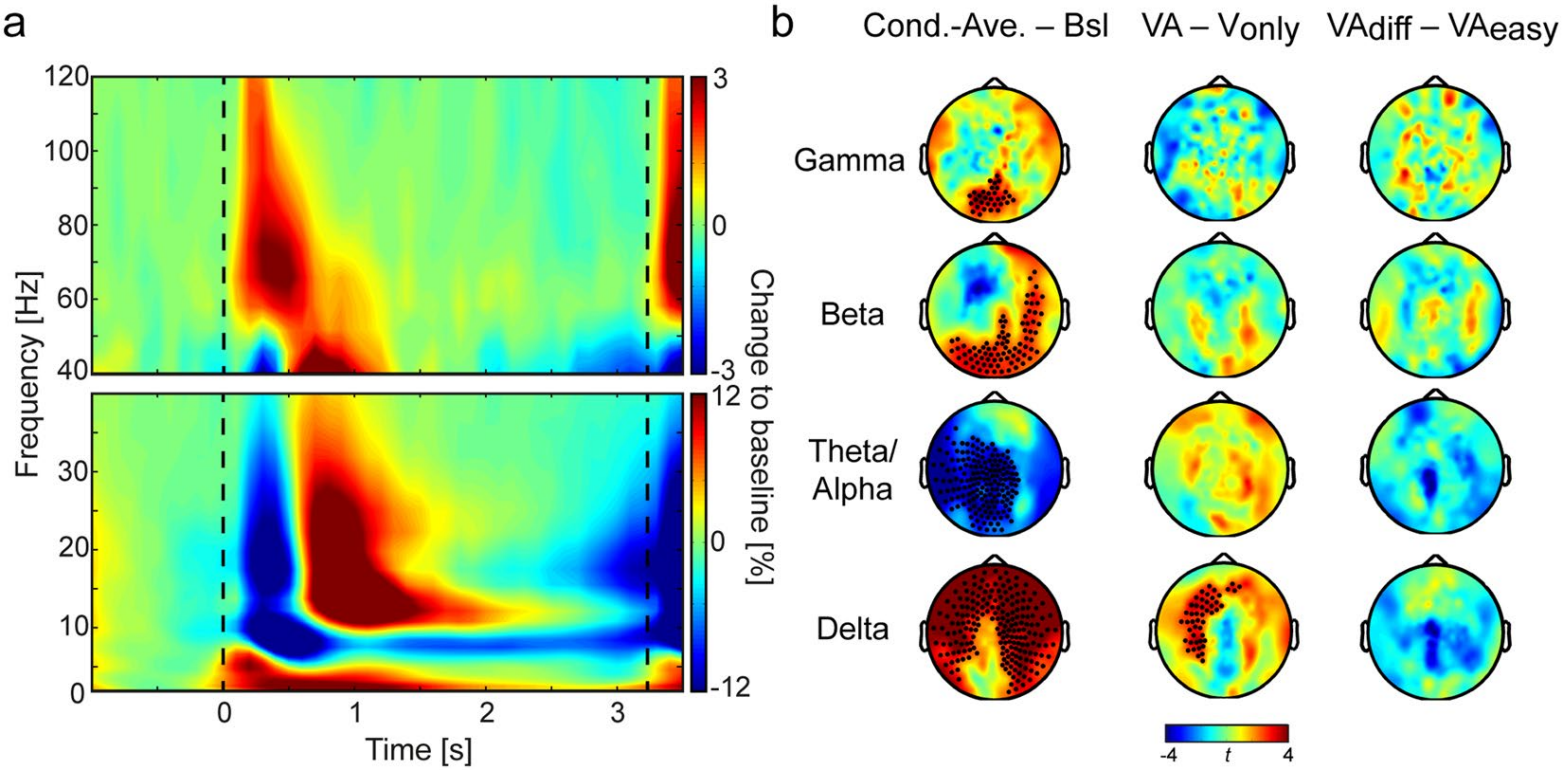
Spectral power results. (**a**) Baseline corrected spectral power in all frequencies averaged across all participants, sensors and conditions (grand average). Warm colours depict enhanced spectral power; cold colours represent reduced power as compared to baseline. Dashed lines mark the onset of the sample (0 ms) and the probe stimuli (3200 ms). Based on significantly modulated power during the delay period (500–3000 ms), we selected frequency-bands-of-interest in the delta, theta/alpha, beta and gamma range (see Supplementary Fig. S1 and main text). (**b**) Frequency-band specific power differences for the three independent contrasts used throughout the study: condition-average vs. baseline; dual-task WM vs. single-task WM; visual + auditory_difficult_ vs. visual + auditory_easy_ WM. Black dots indicate clusters of sensors with significant differences for the considered contrast. The only difference we found between the three conditions was apparent in the delta-band, where power was enhanced over left frontal and central sensors during dual-task as compared to single-task WM maintenance.

In order to define frequency-bands-of-interest independent of location and condition contrasts for our spectral power analysis, we first tested power averaged across all sensors and conditions for all time-frequency bins between 0 (sample stimulus onset) and 3000 ms against a pre-stimulus baseline window (see Supplementary Fig. S1; all clusters with p_corr_ < 0.01). Figure 2a shows spectral power modulations averaged across all sensors, conditions, and participants during the whole trial. Since the results highly resembled our findings from our earlier study^9^, in the current study we selected similar frequency bands to enhance comparability. In our earlier study, however, the low beta band (13.7–17.5 Hz) turned out to show no differences between the memory and the non-mnemonic control condition and most likely was not specific to WM maintenance. Therefore, we omitted this frequency band for the analysis of the data in the current study. Furthermore, in contrast to our earlier study, frequencies in the delta range also showed modulated power throughout the whole delay period and were therefore added to the frequency-bands-of-interest. Hence, in the current study we selected the following frequency bands for further spectral power analysis: delta (2.0–4.1 Hz), theta/alpha (6.7–8.5 Hz), beta (19.7–31.9 Hz), and gamma (40.5–94.3 Hz).

Figure 2b shows differences in spectral power between the averaged delay periods and the baseline window (left column) as well as differences within the delay period in each of the defined frequency bands for the two pre-defined contrasts-of-interest: *dual-task* minus *single-task* (VA − V_only_, middle column) as well as *visual* + *auditory*_*difficult*_ minus *visual* + *auditory*_*easy*_ (VA_diff_ − VA_easy_, right column). The averaged conditions showed elevated broadband gamma power during the delay period as compared to the baseline window in a cluster of occipitoparietal sensors. However, no significant difference in gamma power was found between the conditions. To examine whether differences between the conditions were already apparent in the baseline and therefore diminished by a condition-specific baseline-correction, we also applied an ANOVA to the power estimates from the baseline window. Again, no significant main effect was found.

Beta amplitudes were elevated during the delay period as compared to the baseline window within a cluster consisting of occipital, temporal, and parietal sensors in the average across the three conditions. However, no significant differences between the conditions in neither the delay nor the baseline periods were apparent in the beta-band.

In the average across all three conditions, amplitudes within the theta/alpha-band were decreased during the delay period as compared to the baseline window in a large network of mostly left posterior, central and temporal sensors. Differences between the conditions were neither found within the delay periods nor the baseline windows.

Delta power was increased mostly over frontal, temporal and central regions between the delay period and the baseline window in the averaged conditions. Differences between the conditions were apparent in increased delta power over left lateralised central and frontal regions in the averaged VA conditions as compared to the V_only_ condition (Fig. 2b, middle column). However, no differences between the two dual-task conditions (VA_diff_ − VA_easy_) were found in the delta-band and there were no differences in the baseline window between the three conditions.

In sum, significant differences in spectral power were restricted to the delay period vs. baseline comparisons in all frequency bands except for the delta-band, where dual-task performance was associated with widespread left-lateralised increased spectral power (see Supplementary Fig. S2 for time-resolved power differences between the condition-averaged delay periods and the baseline in all frequency bands).

### Phase-amplitude coupling

We computed local PAC during the delay periods and the baseline windows of the three conditions for a variety of low- and high-frequency band combinations at each individual sensor. Cluster-based permutation statistics was then employed to identify clusters of sensors and frequencies showing significant differences either between the delay periods and the baseline windows (averaged across conditions) or within the delay periods between the conditions.

**Figure 3:**
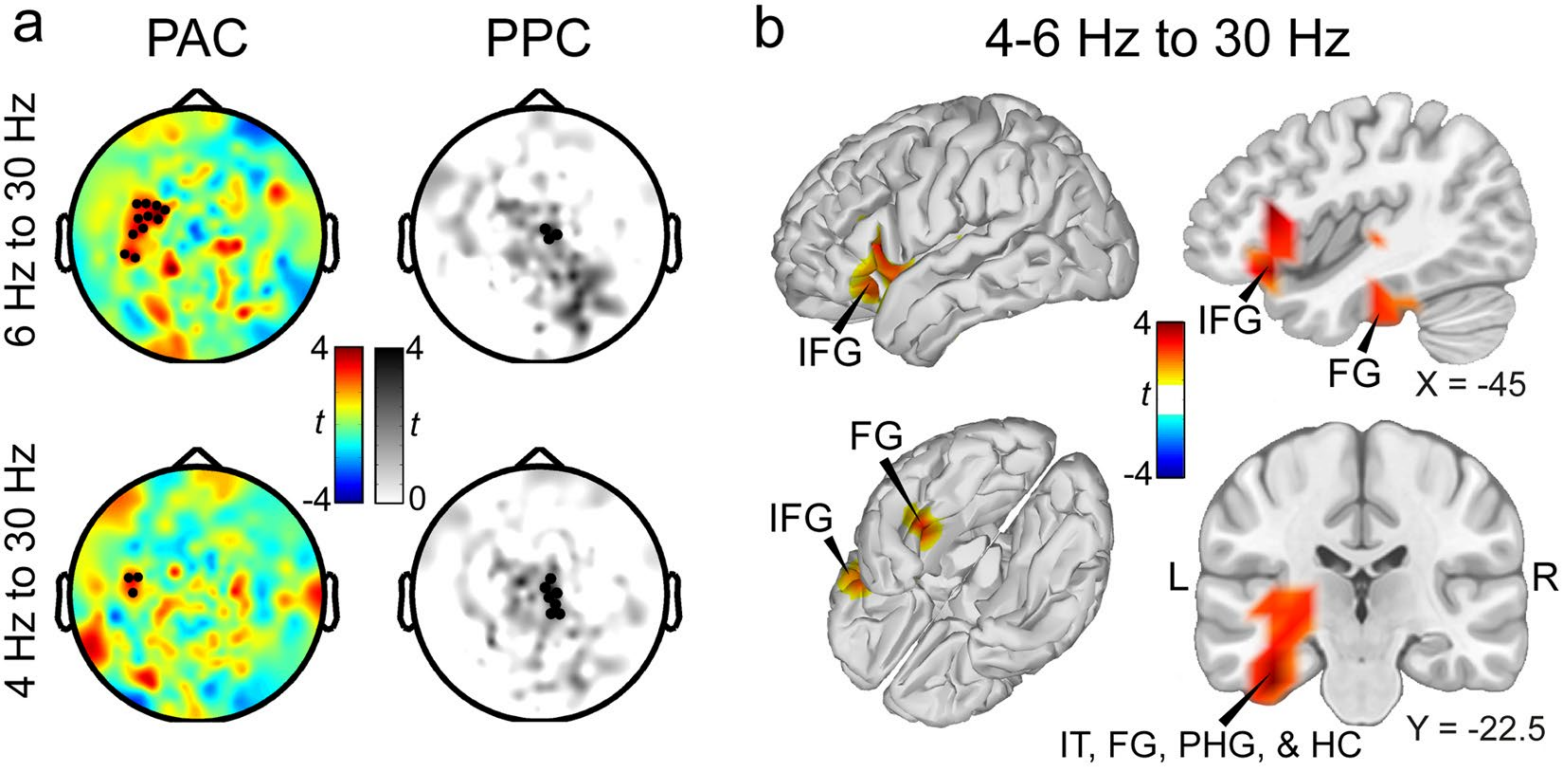
Cross-frequency phase-amplitude and phase-phase coupling during the averaged delay periods as compared to baseline. (**a**) Cluster-based permutation statistics revealed a cluster of sensors showing significantly enhanced PAC between the phases of frequencies from the theta range and the amplitudes of frequencies from the beta range during the averaged delay periods as compared to the baseline windows. No further clusters were found between other frequencies or in the other two contrasts. To investigate whether the effect found for PAC was accompanied by an effect in PPC, which would hint towards waveform-dependent cross-frequency coupling^25,29^, we computed PPC for the frequencies of the PAC effect. A cluster of right central sensors displayed enhanced PPC during the delay period compared to baseline (black dots). Importantly, the cluster did not include sensors of the cluster found for PAC. (**b**) Source analysis of the PAC effect found on sensor level revealed a cluster of voxels from MTL and IFG with significantly enhanced PAC during the delay period. Coordinates are given in MNI space. Abbreviations: FG: fusiform gyrus; IFG: inferior frontal gyrus; IT: inferior temporal cortex; HC: hippocampus; L: left; PAC: phase-amplitude coupling; PHG: parahippocampal gyrus; PPC: phase-phase coupling; R: right.

Employing cluster-based permutation statistics resulted in one cluster including left temporal and frontal sensors. In this cluster, the phases of the centre frequencies at 4 and 6 Hz and the amplitudes of the centre frequency at 30 Hz showed differences in PAC between the across-conditions averaged delay periods and the baseline windows (Fig. 3a; *p*_*corr*_ < 0.01; see Supplementary Fig. S3 for a comodulogramm showing PAC differences for all considered frequency pairs as well as depictions of beta power distributions across the theta phase for each participant). Apart from this theta-beta PAC, no other low/high frequency pair revealed significant effects. Furthermore, no differences were found between the conditions during the delay periods.

Recently, several concerns have been raised that PAC between two frequency bands might be dependent on the non-sinusoidal waveform shape of the low frequency oscillation rather than on an interaction between two distinct neural processes that are represented by oscillations at different frequencies^24–28^. One indication that the observed effect in PAC is dependent on a non-sinusoidal waveform rather than representing an interaction between two distinct processes, would be a co-occurrence of significant cross-frequency phase-phase coupling (PPC)^25,29^. To check whether our effect in theta-beta PAC is accompanied also by a significant change in PPC, we computed PPC between the phases of the 4 and 6 Hz and the phase of the 30 Hz signals (Fig. 3a). Employing cluster-based permutation statistics for PPC between the frequency combinations, we found a cluster of right central sensors showing significantly enhanced PPC in the averaged delay periods as compared to baseline (*p*_*corr*_ < 0.05; Fig. 3a). Importantly, none of the sensors showing significantly enhanced PPC were included in the cluster of sensors showing significantly enhanced PAC. No further clusters of enhanced or reduced PPC were found. Moreover, we tested for significant differences in PPC specifically within the cluster of sensors showing enhanced PAC. A two-sided paired *t*-test on the average across the sensors included in the PAC cluster yielded no significant differences in PPC between the averaged delay periods and the baseline window (*t*_(26)_ = 0.53; *p* = 0.60; *d* = 0.10).

To identify the sources of the PAC effect found on sensor level, we projected the data to source space and computed PAC between the phases of the 4 and 6 Hz frequency bands and the amplitudes of the 30 Hz frequency band during the delay periods and baseline windows of all conditions in all voxels. Employing cluster-based permutations statistics, we found a cluster of voxels spanning from left IT, hippocampus (HC), parahippocampal gyrus (PHG), and fusiform gyrus (FG) over Heschl’s gyrus to inferior frontal gyrus (IFG) showing significant differences between the delay periods and the baseline (Fig. 3b; *p*_*corr*_ < 0.01; see Fig. S4 for unmasked, whole-brain differences in theta-beta PAC).

Differences in spectral power in the low frequency band can bias PAC results due varying signal-to-noise ratio and the associated differences in phase estimation quality between the conditions. We therefore tested for differences in spectral power within the sensors and voxels showing differences in PAC in the low frequency band involved in PAC. There was no significant difference between the averaged delay periods and the baseline window in the whole low frequency band involved in PAC (i.e. 3–7 Hz; sensors: *t*_(26)_ = 0.57; *p* = 0.57; *d* = 0.11; voxels: *t*_(26)_ = 1.55; *p* = 0.13; *d* = 0.30) nor in the low frequency band showing the stronger PAC effect (i.e. 5–7 Hz; sensors: *t*_(26)_ = −1.46; *p* = 0.16; *d* = −0.28; voxels: *t*_(26)_ = 0.34; *p* = 0.73; *d* = 0.07). Amplitudes in the higher frequency bands were normalized before computing PAC. Hence, differences in spectral power between the delay period and the baseline in the beta-band would not bias our results for PAC.

### Long-range phase synchronisation

**Figure 4:**
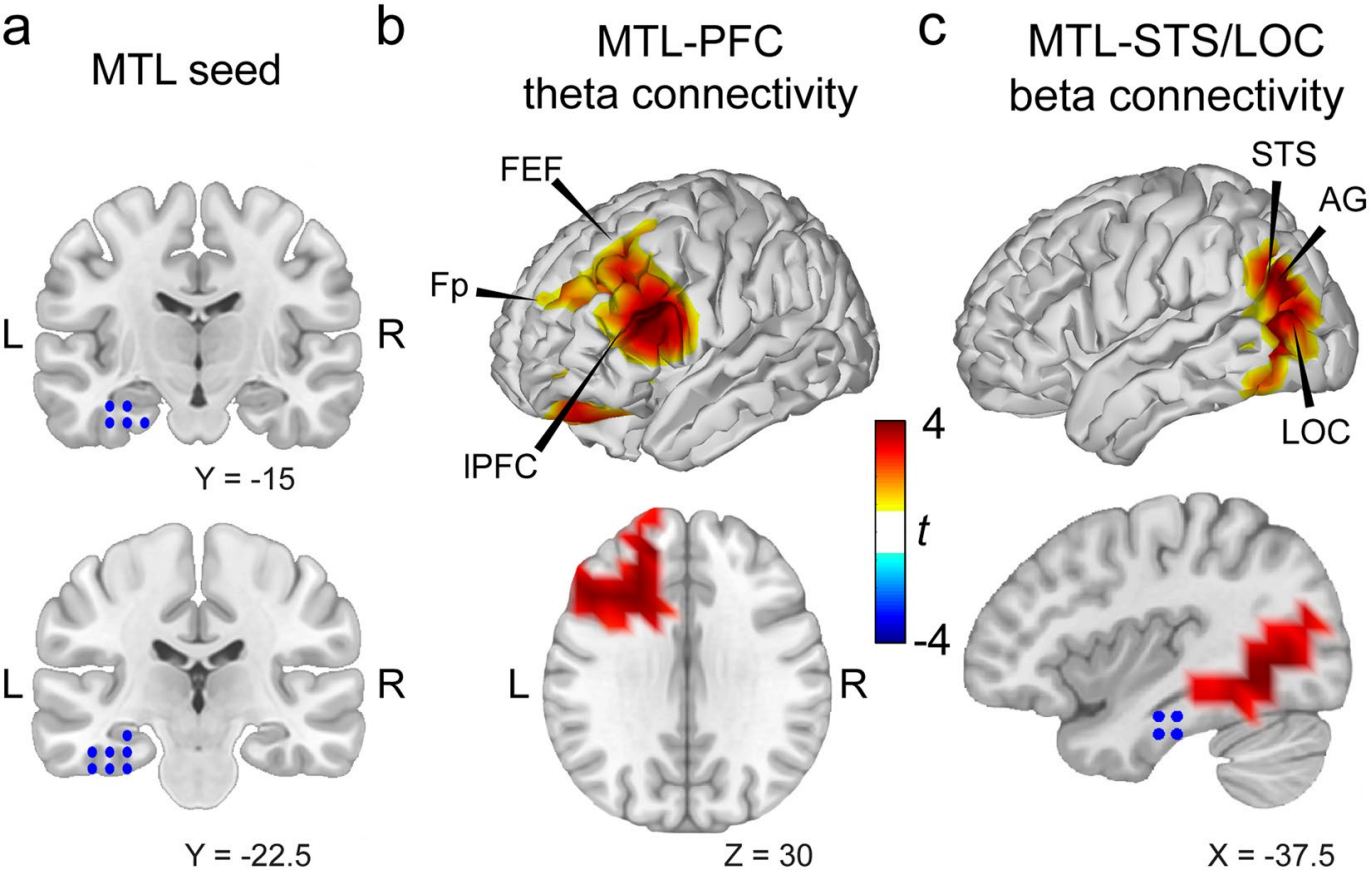
Long-range phase synchronisation during audiovisual as compared to visual WM from MTL seed voxels for the frequency bands involved in PAC. (**a**) Voxels showing significant PAC were selected as seed region for the whole-brain phase synchronisation analysis within the two frequency bands. Coordinates are given in MNI space. (**b**) Cluster of voxels in lPFC showing enhanced imaginary coherence with the MTL seed region within the theta-band involved in PAC during the dual-task as compared to the single-task WM condition. No further clusters were found. (**c**) Cluster of voxels in visual and audiovisual areas, including STS, AG, and LOC, showed enhanced imaginary coherence with the MTL seed region within the beta-band involved in PAC during the dual-task as compared to the single-task WM condition. Again, no further clusters were found. Abbreviations: AG: angular gyrus; FEF: frontal eye fields; Fp: frontopolar cortex; L: left; LOC: lateral occipital complex; lPFC: lateral prefrontal cortex; MTL: medial temporal lobe; R: right; STS: superior temporal sulcus.

To investigate whether the brain regions exhibiting significant local PAC were connected to distant areas via phase synchronisation, we analysed connections to all other voxels in the brain within the low and the high frequency bands involved in theta-beta PAC. Based on earlier results showing modulated PAC in MTL regions during visual WM^9,20,30^ as well as findings related to auditory and audiovisual WM maintenance in IFG regions^31^, we split the PAC-region into two seed regions for the phase synchronisation analysis. One region consisted of only MTL voxels from left IT, HC, PHG, and FG (Fig. 4a). The other region included the anterior voxels from IFG and Heschl’s gyrus. Since spectral power in the delta-band (2.0–4.1 Hz) differed in frontal regions between the conditions, we restricted the analyses of coherency in the low frequency band (theta-band) to frequencies between 4.5 and 7 Hz (instead of 3–7 Hz; 4 frequency bins) in order to avoid differences in signal-to-noise ratios between the conditions. For analyses of coherency in the high frequency band, we used the beta-band already selected for the spectral power analysis, which included the amplitude frequencies from the PAC result (5 frequency bins). Further, we only considered the imaginary part of coherency (imaginary coherence) to avoid spurious coupling due to volume conduction^32^. Cluster-based permutation statistics was employed in order to test for differences in imaginary coherence between the conditions during the delay period. Testing connections from the MTL seed region (Fig. 4a) to all other voxels in the brain within the theta-band, we found a cluster of PFC voxels showing a significant difference between the averaged dual-task and the single-task conditions (*p*_*corr*_ < 0.01; Fig. 4b). The cluster included voxels from lateral PFC (lPFC), frontopolar cortex (Fp), and frontal eye fields (FEF). In the beta-band, we found a cluster of temporooccipital voxels with significantly enhanced imaginary coherence in the VA conditions as compared to the V_only_ condition (*p*_*corr*_ < 0.01; Fig. 4c). Here, the cluster included voxels from left FG, posterior inferior and superior temporal sulcus (STS), angular gyrus (AG), and lateral occipital complex (LOC). No differences were apparent for the condition contrast VA_diff_ minus VA_easy_ and there were also no differences for the analysis of the frontal seed region (IFG) in any of the two frequency bands.

Since power differences can drive results in coherency analysis, we specifically tested for differences in theta and beta power for both, the seed regions as well as the coupled regions. We did not find significant differences in power in any of the considered regions in neither the theta band (MTL seed region: *t*_(26)_ = 0.63; *p* = 0.54; *d* = 0.12; PFC cluster: *t*_(26)_ = 0.29; *p* = 0.78; *d* = 0.06) nor in the beta band (MTL seed region: *t*_(26)_ = −1.25; *p* = 0.22; *d* = −0.24; temporooccipital cluster: *t*_(26)_ = 0.42; *p* = 0.68; *d* = 0.08; see Fig. S5 for whole-brain differences in theta and beta power).

Finally, we tested whether the significant connections between MTL and PFC voxels within the theta-band as well as between MTL and temporooccipital cortex within the beta-band showed a preferred direction of information flow. Therefore, we computed the phase-slope index (PSI)^33^ for every connection within the cluster of significant voxels in each frequency band and tested the average across voxels from the VA conditions to the average from the V_only_ condition. However, no preferred direction in the flow of information was apparent in the theta-band (*t*_(26)_ = −0.55; *p* = 0.59; *d* = −0.11), nor in the beta-band (*t*_(26)_ = 1.55; *p* = 0.13; *d* = 0.30).

## Discussion

We used MEG to record neural oscillatory activity from participants performing audiovisual delayed-match-to-sample tasks (Fig. 1a). Our first goal was to manipulate top-down cognitive control during WM maintenance by asking participants to (a) respond to visual stimuli only or (b) to follow dual-task instructions requiring the retention of visual and auditory stimuli in separate conditions of the experiment. Furthermore, we aimed at investigating the neural correlates of domain-specific WM resources by modifying the auditory task difficulty. We found that WM maintenance was associated with enhanced theta-beta cross-frequency PAC in MTL as well as IFG regions (Fig. 3b). During enhanced top-down control in the dual-task conditions (VA) as compared to the single-task (V_only_), these MTL regions exhibited increased phase synchronisation to frontal regions within the theta-band (Fig. 4b). Such an increase in frontotemporal theta phase synchronization strongly supports the view that enhanced cognitive control during audiovisual WM maintenance is associated with enhanced long-range frontotemporal phase synchronization. Moreover, the same contrast revealed enhanced phase synchronisation within the beta-band between MTL regions and more posterior visual and audiovisual areas such as STS and LOC (Fig. 4c). This increase in synchronized beta activity between STS, LOC and MTL regions might be associated with the maintenance processes of the audiovisual information during the dual-task WM conditions.

The spectral power differences that we observed between the averaged WM delay periods and the baseline windows (Fig. 2; Supplementary Fig. S2) are consistent with earlier reports of modulated power during visual WM maintenance^9,14,15,18,34^. In an earlier study using the same visual stimulus material, we compared visual WM maintenance processes against a non-mnemonic control condition, in which stimulus-driven bottom-up processes were kept comparable between the conditions^9^. The spectral power differences within the theta/alpha, beta and gamma-band found during the WM delay periods in the current study strongly resemble the differences in spectral power within these frequency bands reported in our earlier work. Both studies show elevated spectral power in the beta- and gamma-band over posterior areas as well as decreased power in the theta/alpha range mostly over left temporal, central and parietal areas (Fig. 2b). It has been argued that power in the beta and gamma range represent neural correlates of active stimulus maintenance processes^18^, while decreased power in the alpha range mirrors a release of inhibition of areas relevant for ongoing WM maintenance^35^. Our results are in close agreement with these hypotheses.

In the current study, we further found enhanced amplitudes for frequencies in the delta-band, which were the only frequencies showing differences in spectral power between the conditions. Delta band power was significantly enhanced in the dual-task WM conditions as compared to the single-task WM condition over frontal and central sensors. An increase in frontal delta power could therefore be associated with enhanced cognitive control processes during audiovisual WM maintenance. However, since these differences were not apparent when comparing WM maintenance against a control task in our earlier study^9^, where task difficulty did not differ between the conditions, we suspect that the increase in delta power might not be specific to WM maintenance per se. Prefrontal delta oscillations have mainly been associated with neural processes of motivation and attention^36^. Enhanced spectral power during dual-task WM might therefore represent a correlate of increased task difficulty and cognitive load during the dual-task WM conditions. However, our data does not allow to disentangle these processes and future research is needed to shed light on a potential link between frontal delta power and multisensory WM maintenance.

Our finding of theta-beta PAC (Fig. 3) is in close agreement with the results from our earlier study. In both experiments, PAC between phases of frequencies from the theta range and amplitudes from the beta range was enhanced during WM maintenance in temporal regions – including IT, an area known to be involved in WM maintenance of visual object information in monkeys^37–39^ as well as humans^30,40^. In the current study, theta-beta PAC was additionally apparent in deeper MTL structures such as the HC, PHG, and FG, which fits to studies reporting PAC as well as within-frequency synchronisation within and between MTL structures during visual WM maintenance^20,30,41,42^. Our result provides additional evidence that PAC might represent a neural marker of sustained WM maintenance taking place in MTL structures^19^.

The cluster of voxels showing enhanced theta-beta PAC during WM maintenance in the current study also included more anterior temporal and frontal areas such as Heschl’s gyrus and IFG (Fig. 3c). Studies investigating WM processes in animals and humans reported areas of the IFG (or homologous in monkeys, the ventral lPFC) to be involved especially in auditory or audiovisual WM maintenance^31,43^. In a recent study by Plakke *et al*.^31^, for instance, areas of the ventral lPFC were inactivated while monkeys performed a visual, an auditory, or an audiovisual WM task. They found that inactivation of these areas caused an impairment of WM maintenance for auditory and audiovisual, but not for visual information alone. Our results of enhanced PAC in IFG might be associated with such WM maintenance processes of the auditory or audiovisual stimulus material. This is in line with the finding that these areas were not involved in WM maintenance in our earlier study, where no auditory stimulus material was utilised^9^.

Our analysis of PPC between the frequencies involved in PAC revealed that sensors exhibiting enhanced PAC during WM maintenance did not exhibit enhanced PPC. If the observed effect in PAC was only dependent on the waveform-shape of the low frequency band, in which case enhanced PAC would be observed between the base frequency and its harmonics^24,25^, then these frequencies should also exhibit enhanced PPC^29^. Moreover, the beta-band exhibited enhanced phase synchronisation from MTL to temporooccipital visual areas. These areas, however, did not reveal any differences in theta/beta-PAC, which suggests that the beta-band represents a distinct neural oscillation, which exists independent of the waveform shape of the theta oscillation.

The similarity between the results reported here and those from earlier studies investigating pure visual WM maintenance as well as the fact that mainly visual areas were involved in maintenance processes in the current study suggests that the differences found in power as well as PAC between the delay periods and the baseline windows mainly represent neural correlates of WM maintenance processes for visual stimulus material. No sustained power effects in higher frequency bands were found over auditory cortices and no differences in WM-related spectral power or PAC were found between the audiovisual and the visual WM conditions. One plausible explanation is that the neural processes specifically associated with auditory WM maintenance were too weak to be picked up by MEG in the current experimental setting. Considering that we only used simple sinusoidal sounds at fixed frequencies as auditory stimulus material, sparse neuronal representations of the sound stimuli might have precluded the chance to detect sustained power differences in the delay periods. Using more complex artificial or natural sounds probably would have improved the signal-to-noise ratio of the ongoing auditory processes. In addition, we cannot exclude that to some extent participants also maintained the task-irrelevant auditory stimuli during the single-task WM condition. In that case, the signal-to-noise ratio in the averaged conditions compared to the baseline windows could have been sufficient to identify regions of ongoing auditory or audiovisual WM maintenance represented by PAC, such as the IFG^31,43^, but too weak to disentangle the differences between the conditions.

Similarly, we also do not find any differences in oscillatory brain activity between the VA_easy_ and the VA_diff_ conditions. Again, one likely reason could be the fact that the sinusoidal sounds were so simple that only very small neuronal assemblies were engaged specifically with auditory or audiovisual processes. Although we expected that higher task difficulty would lead to increased demands on top-down control mechanisms, the results indicate that in our paradigm these demands might not be reflected in differential oscillatory activity during the maintenance interval. In contrast, differences between the easy and difficult conditions could probably only have arisen at the time of stimulus retrieval, when the probe had to be matched to the sample stimulus.

The lack of differences between the three conditions in accuracies for the visual stimuli, spectral power over visual sensory areas, and PAC in MTL structures strongly indicates that visual WM maintenance processes were not affected by an additional auditory WM task. This would be in line with earlier studies suggesting domain-specific resources for auditory and visual WM processes, which are not shared across the modalities^44–46^. However, an alternative view would be that enhanced cognitive control over visual maintenance processes during dual-task WM allowed for equalized performances of the visual WM task, causing also equalized levels of accuracies, power, and PAC between the conditions. In such case these measures would be in fact not well suited to depict the modulated processes between the conditions. Cognitive control processes during WM maintenance and other cognitive processes have been associated with long-range phase synchronisation especially in the theta-band^7–9,11^. Therefore, according to this alternative view, enhanced phase synchronisation in the theta-band between frontal and temporal areas involved in WM maintenance would be apparent. In fact, our finding of increased phase synchronisation in the theta-band between PFC and MTL is strongly in favour of this hypothesis. It indicates that prefrontal top-down control might be exerted over areas where ongoing WM maintenance processes take place, which presumably are represented by enhanced PAC. This provides further evidence that the interplay of top-down cognitive control and local WM maintenance processes might be depicted by the combination of long-range phase synchronisation and local PAC. Such combination could represent a mechanism for long-range neuronal communication across distant brain sites and frequencies^9,47–49^. However, in the current study we do not find any evidence for a preferred direction of information flow from one region to the other, leaving interpretations regarding directed communication unwarranted.

Lastly, dual-task versus single-task performance was also associated with enhanced phase synchronisation in the beta-band between MTL and posterior visual and audiovisual areas including STS and LOC (Fig. 4c). This beta phase synchronisation effect underscores the central role beta-band oscillations presumably play for the maintenance of visual object representations in the ventral stream. Studies using functional magnetic resonance imaging^50,51^ have linked the LOC to processes of object recognition. Our finding is in agreement with earlier studies associating beta phase synchronisation among temporooccipital areas with WM maintenance^52^ and the hypothesis that synchrony in the beta-band is elevated as long as a current cognitive state is maintained^53^. Moreover, since also the STS, an area known to be involved in audiovisual integration processes^54^, showed enhanced phase synchronisation with MTL, this result might depict the maintenance processes of the integrated audiovisual information during the dual-task WM conditions. However, as stated above, more complex sounds might be helpful to further investigate the WM maintenance processes of auditory or audiovisual information.

In conclusion, our results suggest that cognitive control of active WM maintenance processes might be governed by low-frequency phase synchronisation between frontal cortex and stimulus-processing temporal brain regions. Enhanced cognitive control required during audiovisual WM maintenance was associated with an increase in theta phase synchronisation from lPFC to MTL regions, where enhanced theta-beta PAC indicated processes of WM maintenance. Here, we provide further evidence that such a combination of long-range phase synchronisation and local cross-frequency PAC might be a promising mechanism for neuronal communication between distant brain regions and across frequencies.

## Methods

### Participants

Twenty-nine healthy participants (age (mean ± SDs): 25.0 ± 3.9 years; 22 females; all right-handed) gave written informed consent to take part in the study. Participation was monetarily compensated with 10 €/hour. All participants reported to have normal audition, normal or corrected-to-normal vision and no background of neurological or psychiatric disorder. To make sure that all stimuli were new to the observers and to avoid potential long-term memory effects, we excluded all participants who already volunteered in the predecessor study^9^. The ethics committee of the Medical Association Hamburg approved the study protocol, and the experiment was carried out in accordance with the approved guidelines and regulations.

### Experimental procedure

In the current study, we used an audiovisual version of a visual delayed-match-to-sample task that we already utilised in an earlier study^9^. The time course of a trial was as follows. After an initial presentation of a light-grey fixation dot for 2500 to 3000 ms, the visual and auditory sample stimuli were presented for 200 ms (Fig. 1a). During the delay period the fixation dot was again presented for 3000 ms followed by the presentation of the visual and auditory probe stimuli for 200 ms. The participants’ task was to judge whether the probe stimuli were an identical or a modified version of the sample stimuli.

We used line drawings of natural objects originally introduced by Snodgrass and Vanderwart^55^ as visual sample stimuli. Visual probe stimuli were identical or modified versions of these sample stimuli^9^. Fourteen different sinusoidal sounds with frequencies between 400 and 500 Hz in steps of 7.5 Hz were used as auditory sample stimuli. Auditory probe stimuli differed in pitch from the sample stimuli (see below).

The current experiment comprised three conditions. In the *visual-only* condition (V_only_), participants were asked to only remember the visual sample stimulus and to compare it to the visual probe stimulus. They were told to ignore the auditory stimulus. Please note that *visual-only* here refers to the task instructions only. Each trial always comprised an auditory and a visual stimulation. In order to assure physically comparable stimulation between the conditions, auditory probe stimuli differed randomly either by ±7 or by ±50 Hz from the sample stimulus in half of the trials. In the other half, probe stimuli did not differ from sample stimuli. In the other two conditions, participants were asked to remember the visual as well as the auditory sample stimuli. Their task was to consecutively judge whether the two probe stimuli were either the same as or modified versions of the sample stimuli. Visual and auditory probe stimuli were always manipulated independently from each other, meaning that modifications to the visual stimuli were not informative of modifications to the auditory stimuli and vice versa. Auditory probe stimuli differed by ±50 Hz from the sample stimulus in the *visual* + *auditory*_*easy*_ condition (VA_easy_), whereas they differed by ±7 Hz in the *visual* + *auditory*_*difficult*_ condition (VA_diff_) in half of the trials. In the other half, sample and probe stimuli had the same frequency.

Conditions were administered block-wise. Participants were informed about the upcoming condition prior to each block and were required to respond as fast and accurately as possible first for the visual and then for the auditory stimuli by pressing a button with the right index or middle finger, respectively. Response mapping and condition sequence were counterbalanced across participants. All other manipulations were fully counterbalanced within each participant. The experiment comprised 15 blocks (5 blocks per condition) with 28 trials per block (420 trials in total). To familiarise participants with the task, a short training session comprising four trials of each of the three conditions was conducted before the recording. Visual training stimuli were not part of the subsequent recording session.

For stimulus presentation, we used Matlab (Version: 8.0, R2012b; MathWorks, Natick, MA; RRID: SCR_001622) and Psychtoolbox^56^ (Version: 3.0.10; RRID: SCR_002881) on a Dell Precision T5500 with a Windows 7 Professional 64-bit operating system. The visual stimuli were projected onto a matte backprojection screen at 60 Hz with a resolution of 1280 × 1024 pixels positioned 65 cm in front of participants. The auditory stimuli were presented at 48 kHz and 80 dB using MEG-compatible in-ear headphones (STAX, SRM-2525).

### Data acquisition and pre-processing

During the experiment, we recorded MEG at a sampling rate of 1200 Hz using a 275-channel whole-head system (Omega, CTF Systems Inc.) situated in a dimly lit, sound attenuated and magnetically shielded chamber. In order to have a better estimate for endogenous artefacts, we recorded electrical eye, muscle and cardiac activity with additional Ag/AgCl-electrodes. By means of online head localisation, participants were navigated back to their original head position prior to the onset of a new experimental block if their movements exceeded five mm from their initial position. Seven malfunctioning channels were excluded from analysis.

We analysed behavioural data using R^57^ (Version: 3.1.1; RRID: SCR_001905) and RStudio (Version: 0.98.978; RStudio Inc., Boston, MA; RRID: SCR_000432). An interpretation of reaction time effects was confounded by the strong differences in task demands between the conditions as well as sequential button presses to the visual and auditory probe stimuli, respectively. Therefore, we refrained from analysing reaction times to either of the button presses. Physiological data analysis was realised in Matlab 2013a utilising the M/EEG analysis toolbox FieldTrip^58^ (Version: 20140413; RRID: SCR_004849) and custom-made scripts. The continuous recording was first cut into epochs of 6.2 s length around the onset of the sample stimulus (−1.5 to 4.7 s relative to sample onset). We used semi-automatic procedures implemented in FieldTrip to reject trials containing jump and strong muscle artefacts. The remaining trials were filtered using a high-pass filter at 0.5 Hz, a low-pass filter at 170 Hz, and three band-stop filters at 49–51, 99–101, and 149–151 Hz. After down-sampling the data to 600 Hz, an independent component analysis (infomax algorithm) was performed allowing for a manual removal of components containing eye-movement, muscle, and cardiac artefacts. These components were identified by visual inspection of their time course, variance across samples, power spectrum, and topography^59^. On average, 22.0 ± 5.4 components were rejected. As a final step, all trials were again visually inspected and trials containing artefacts that were not detected by the previous steps were removed. 394.4 ± 16.0 trials of a total of 420 trials remained from pre-processing on average.

### Data analysis

The analysis procedure is outlined in the following. (1) In order to define contrast-independent frequency-bands-of-interest, we first tested the average of spectral power across all channels and conditions (grand average) in every time-frequency bin against a pre-stimulus baseline window (average from −800 to −300 ms) at the group-level. Based on significantly modulated spectral power during the delay period (500 to 3000 ms), we selected frequency-bands-of-interest for further spectral power analyses. (2) For each frequency band, we tested spectral power averaged across the delay period of all conditions against the baseline window in every channel. Two more contrasts were then applied to test for potential spectral power differences between the conditions during the delay period: dual-task minus single-task (i.e. averaged visual + auditory conditions minus visual only condition; VA − V_only_) and visual + auditory_difficult_ minus visual + auditory_easy_ (VA_diff_ − VA_easy_). (3) Local PAC was computed for different combinations of low-frequency phase and high-frequency amplitude for data from the baseline window as well as from the delay period in every condition and channel. Cluster-based permutation statistics was applied to test for differences in local PAC between the delay period and the baseline window for an average across all conditions as well as differences within the delay period between the conditions. Computations of local PAC in source space were then based on frequency combinations displaying significant differences on sensor level and cluster-based permutation statistics was again employed to reveal clusters of voxels showing significant differences in local PAC. (4) Based on the source level results from (3), we defined cortical regions showing significant PAC differences as seed regions and analysed long-range phase synchronisation from these areas to all other voxels in the brain during the delay period for the low and the high frequency band involved in PAC. We applied two paired *t*-tests to test for differences between the conditions. (5) To address the direction of information flow, we lastly computed the phase-slope index (PSI)^33^ between areas exposing significant connections via phase synchronisation. Details of each analysis step are provided in the following.

#### Spectral Power

Time-frequency decompositions of MEG recordings were realised using wavelet convolution in the frequency domain. Time series of each trial and channel were convoluted with 35 logarithmically spaced Morlet wavelets between two and 120 Hz. Each wavelet consisted of seven cycles. Event-related fields computed for each condition and participant were subtracted prior to wavelet convolution to consider induced spectral power estimates only^60^. Single-trial power estimates were then averaged within each condition. For all analyses, we considered correct trials only. Hence, to account for differences in trial count between the conditions, we stratified trial numbers per participant by randomly selecting as many trials for each condition as the number available from the condition with lowest accuracy. Please note that in the dual-task conditions we only considered trials, in which answers to both stimuli (visual and auditory) were correct. Hence, on average 78.82 ± 13.94 trials were considered per participant in each condition, with the lowest number being 53 trials. We averaged data into time bins of 100 ms and all time-frequency bins following the sample stimulus onset were normalised using a frequency specific baseline window with data averaged from −800 to −300 ms relative to sample onset.

In order to define frequency bands of interest independently from location and condition we averaged data across all channels and conditions within each participant. Then all time-frequency bins between 0 and 3000 ms were tested against the pre-stimulus baseline window (paired *t*-tests). Employing cluster-based permutation statistics as implemented in FieldTrip^61^ controlled for multiple comparisons. First, neighbouring time-frequency bins displaying an uncorrected *p*-value below 0.05 (two-sided) are combined into clusters. In each cluster, the sum of *t*-values is computed. Through permutations of data across participants (1000 permutations) a null-distribution is created, defining the maximum cluster-level test statistics and corrected *p*-values for each cluster. Significance level was set to α = 0.05 (two-sided).

Power differences between the delay period (500 to 3000 ms) and the baseline window were also tested utilising cluster-based permutation statistics. In this case, data from all conditions were averaged within the defined frequency-bands-of-interest and across all time bins within the delay period and tested against the baseline window in the same frequency band. Neighbouring sensors falling below an uncorrected *p*-value of 0.05 built clusters.

Differences in spectral power between the conditions were tested using two paired *t*-tests. Data were again averaged within the selected frequency bands and the delay period and cluster-based permutation statistics was employed to correct for multiple comparisons. One test was performed on the contrast *dual-task* (i.e. averaged across VA_easy_ and VA_diff_) minus *single-task* WM (VA − V_only_). The other test was performed on the contrast *visual* + *auditory*_*difficult*_ minus *visual* + *auditory*_*easy*_ WM (VA_diff_ − VA_easy_). Clusters with a *p*_*corr*_ < 0.01 were considered significant.

To find out whether differences between the conditions were apparent in the baseline window, we performed a one-way ANOVA with the factor *Condition* within each frequency band for data from the baseline period. Also for this analysis, cluster-based permutation statistics was employed to correct for multiple comparisons.

#### Phase-amplitude coupling

To compute non-linear dependencies between the phase of low frequencies and the amplitude of high frequencies, we computed the modulation index (MI) as introduced by Tort *et al*.^62^. In this procedure, first the instantaneous phase series of a band-pass filtered low-frequency signal and the instantaneous amplitude series of a band-pass filtered high-frequency signal are aligned. Then, the phases of the low-frequency signal are grouped into bins of 20° steps (18 bins) and the amplitudes of the high-frequency signal are averaged within each phase bin. If PAC is evident, the distribution of amplitudes across the phase bins should deviate from a uniform distribution. In contrast, if amplitudes are uniformly distributed across the phase bins, the amplitude of the high-frequency signal does not depend on the phase of the low-frequency signal. Deviations from a uniform distribution are defined with a discrete and normalised version of the Kullback-Leibler divergence, i.e., the MI.

We investigated differences in PAC of several frequency pairs during the delay period between the three conditions, and also between the condition-averaged delay period and a baseline period. Due to the restricted amount of data available in the baseline period (500 ms) and the resulting differences in time length compared to the much longer delay period (2500 ms), we avoided biases in signal-to-noise ratio by binning the delay period into time steps of 500 ms. In each time bin, the MI was computed separately. We first cut each time bin of interest from every correct trial, adding a “buffer” window of 200 ms at the beginning and end of each time bin. Then, the raw data was band-pass filtered in the considered frequency bands trial-wise (see below) and a Hilbert transform was computed to extract the instantaneous phase and amplitude, respectively. Subsequently, the buffer windows were removed and the time bins from each trial were concatenated. This procedure avoided filter artefacts at the edges and prevented finding spurious PAC at sharp edges between trials^24^. The concatenated time series were used to compute the MI between the different band-pass filtered signals in each condition separately.

We computed the MI between all combinations of phases from frequencies between 4 and 10 Hz (2 Hz steps/2 Hz bandwidth; termed *phase frequencies*) and amplitudes from frequencies between 18 and 120 Hz (4 Hz steps/adapted bandwidth; termed *amplitude frequencies*) in all sensors. Bandwidth of band-pass filters for amplitude frequencies were adapted to the value of the corresponding phase frequency by using the value of the phase frequency itself as bandwidth.

To test differences in PAC statistically, we first averaged all time bins within the delay period for every sensor. We then tested (a) the condition-averaged delay period against the baseline window; (b) the dual-task against the single-task WM conditions; and (c) the visual + auditory_difficult_ against the visual + auditory_easy_ WM conditions using paired *t*-tests. For all tests, multiple comparisons were again corrected for by employing cluster-based permutations statistics as described above (see *Spectral Power*). Here, neighbouring sensors, phase, and amplitude frequencies displaying a *p*-value below 0.05 were grouped into clusters. Clusters with a *p*_*corr*_ < 0.01 (two-sided) were considered significant.

To assess whether differences in PAC observed for any of the considered frequency pairs were accompanied by differences in cross-frequency phase-phase coupling (PPC), we computed the MI also for phase-phase relationships between frequency pairs that showed significant differences in PAC. To do so, we used exactly the same procedure as described above for PAC, but extracted the phases (in degrees) instead of the amplitudes from the considered high frequency signal in every channel and averaged the phases of the high frequency signal within each phase bin of the low frequency signal. If there is no relationship between the phases of the low and the high frequency pair, then the phases of the high frequency signal should average to around 180° in each phase bin of the low frequency signal, resulting in a uniform distribution across the phase bins. We again computed the distance from such a uniform distribution using the Kullback-Leibler divergence as described above. Statistical tests were performed using the cluster-based permutation procedure for the contrasts that showed significant differences in PAC.

PAC in source space was only computed for frequency combinations that showed significant differences on sensor level. To avoid biases of source projections, common spatial filters containing data from all time steps, conditions, and the considered combination of low- and high-frequency bands were computed for each participant. First, raw time series from all trials were band-pass filtered at the considered frequency bands and Hilbert transformed separately. The cross-spectral density (CSD) matrix was then computed from all trials, time steps, and the two frequency bands, and used to construct a common adaptive linear spatial filter (DICS beamformer)^63,64^ pointing into the direction of maximal variance. Leadfields were based on individual magnetic resonance images and computed using the single-shell volume conductor model^65^ with a 5003 voxel grid that was aligned to the MNI152 template brain (Montreal Neurological Institute, MNI; http://www.mni.mcgill.ca). All trials were finally projected to the 5003 voxels, separately for the low and the high frequency band. In source space, 500 ms time bins were cut from the data (as described above) and concatenated among trials from the same condition. From here, the MI was calculated as described above. This procedure was repeated for every frequency combination involved in significant PAC differences on sensor level.

Frequency combinations belonging to the same cluster on sensor level were then averaged (see *Results*). Statistical tests were performed using paired *t*-tests and multiple comparisons were corrected for by utilising cluster-based permutation statistics with voxel space as the only dimension (uncorrected cluster α = 0.05). Anatomical labeling of voxels in source space was performed using the NFRI functions, a set of Matlab functions described by Singh *et al*.^66^, which makes use of the Automated Anatomical Labelling atlas^67^ as well as the automated Talairach Atlas labels^68^ (http://www.jichi.ac.jp/brainlab/tools.html).

#### Source coherency and phase-slope index

We computed coherency in source space during the delay periods within the frequency bands that were involved in significant modulations in PAC. The localised source of PAC served as a seed region, from which coherency was computed to all other voxels in the brain (see *Results*). CSD matrices were computed from the wavelet convolution results described above between 500 and 3000 ms in each condition and each considered frequency bin within the low- and the high-frequency band. We constructed common and frequency-specific DICS beamformer filters and projected the CSD matrices to source space (details on the forward model are described in the *Phase-amplitude coupling* section). For each frequency bin, coherency was then computed from every seed voxel to all other voxels in the 5003-voxel grid and finally averaged across frequency bins within each band. Since coherency is non-Gaussian distributed, we applied Fisher’s Z transform to all coherency values^32^. For further analyses, we considered the imaginary part of coherency (termed imaginary coherence) only, which is exclusively sensitive to non-zero phase lag connectivity and therefore avoids spurious coupling due to volume conduction^32^.

For statistical comparisons, we averaged imaginary coherence across all considered seed voxels within each condition, resulting in three values for each voxel per participant and per frequency band – each estimating the connectivity between the seed region and that voxel in each condition. Two paired *t*-tests were performed in every voxel to determine differences between the conditions again for the contrasts VA − V_only_ and VA_diff_ − VA_easy_. In all comparisons, cluster-based permutations statistics with voxel space as the only cluster dimension served as multiple comparison correction (uncorrected cluster α = 0.01).

Further, we addressed the directionality of information flow within clusters of significant connections by computing the PSI^33^ for each of the considered conditions. The PSI was first computed for each voxel pair within the cluster showing significant differences in imaginary coherence by using the complex coherency values within the frequency-band-of-interest. Subsequently, we averaged the PSI across all voxel pairs within the cluster and tested differences in directionality between the considered conditions (paired *t*-tests).

### Data availability

The datasets generated and analysed during the current study are available from the corresponding author on reasonable request.

## Electronic supplementary material

Supplementary information
