# Supplementary material for: Cognitive control during audiovisual working memory engages frontotemporal theta-band interactions
Source: Sci Rep. 2017 Oct 3;7:12585. doi: 10.1038/s41598-017-12511-3 (PMC5626716; doi:10.1038/s41598-017-12511-3)
Supplement: Supplementary file 1 — Supplementary information [file 41598_2017_12511_MOESM1_ESM.pdf]

Supplementary Information

Cognitive control during audiovisual working memory engages frontotemporal theta-band interactions

Jonathan Daume[a]*, Sebastian Graetz[b], Thomas Gruber[b], Andreas K. Engel[a], and Uwe Friese[a,c]

[a]University Medical Center Hamburg-Eppendorf, Department of Neurophysiology and Pathophysiology, D-20246 Hamburg, Germany
[b]Institute of Psychology, D-49069 University of Osnabrück, Germany
[c]Institute of Cognitive Science, D-49090 University of Osnabrück, Germany

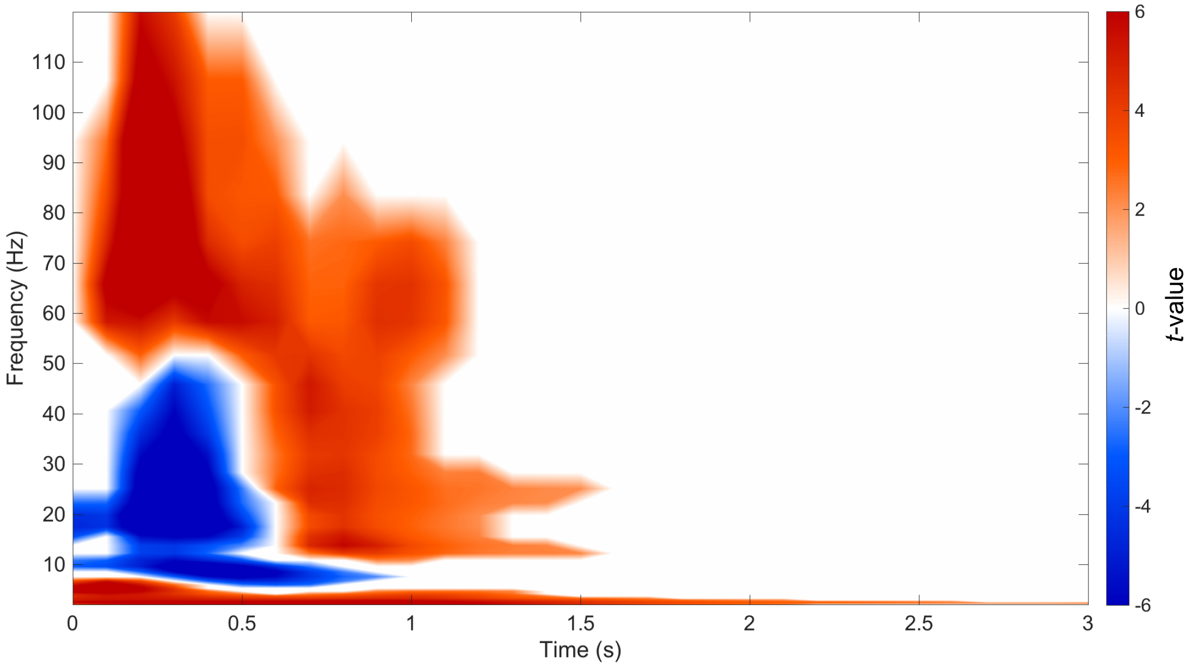

Figure S1. Significantly modulated grand average power as compared to baseline. Spectral power averaged across sensors and conditions (grand average) was tested against a baseline window in all frequencies and time steps between 0 and 3000 ms using cluster-based permutation statistics. Warm colours depict enhanced spectral power; cold colours represent reduced power as compared. Based on significantly modulated power during the delay period (500 – 3000 ms) as well as the findings of our earlier study[1] (see main text), we selected frequency-bands-of-interest in the delta, theta/alpha, beta and gamma range.

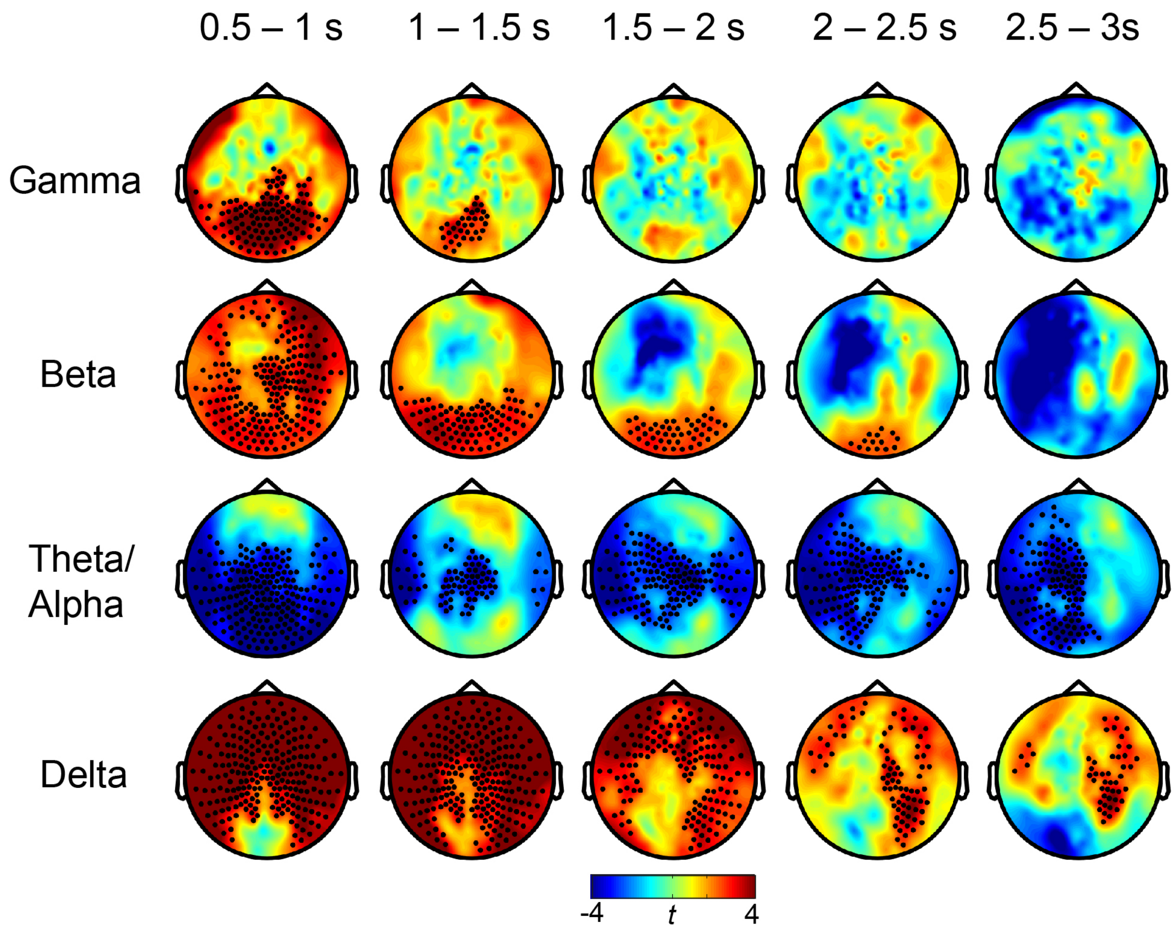

Figure S2. Time-resolved spectral power differences during the averaged delay periods as compared to baseline. Spectral power averaged across all conditions (condition average) was tested against a baseline window in each selected frequency-band-of-interest and all time windows between 500 and 3000 (in 500 ms steps) using cluster-based permutation statistics. Warm colours depict enhanced spectral power; cold colours represent reduced power as compared. As opposed to our earlier study[1], gamma power went back to baseline already before the end of the delay period. However, in contrast to our earlier study, in the current experiment we did not utilize a control condition in which all non-mnemonic components were kept equal to the memory condition. Here, we only compared power to a short baseline window prior to stimulus onset. It seems likely that also in the current study testing the delay periods against a non-mnemonic control period would yield the similar sustained effects as observed in our earlier study. For example, the beta decrease observed over central areas

contralateral to the response hand very likely represents motor preparation processes prior to motor execution. Moreover, decreased gamma and beta power in the latest time window (2.5 – 3 s) might be associated with expectation processes to the upcoming probe stimuli. These processes were also inherent in the control condition in our earlier study and therefore did not appear in the difference plot between the conditions. However, in the contrast used in the current study (delay vs. baseline), these processes might have overshadowed the sustained increases in beta and gamma power in posterior channels.

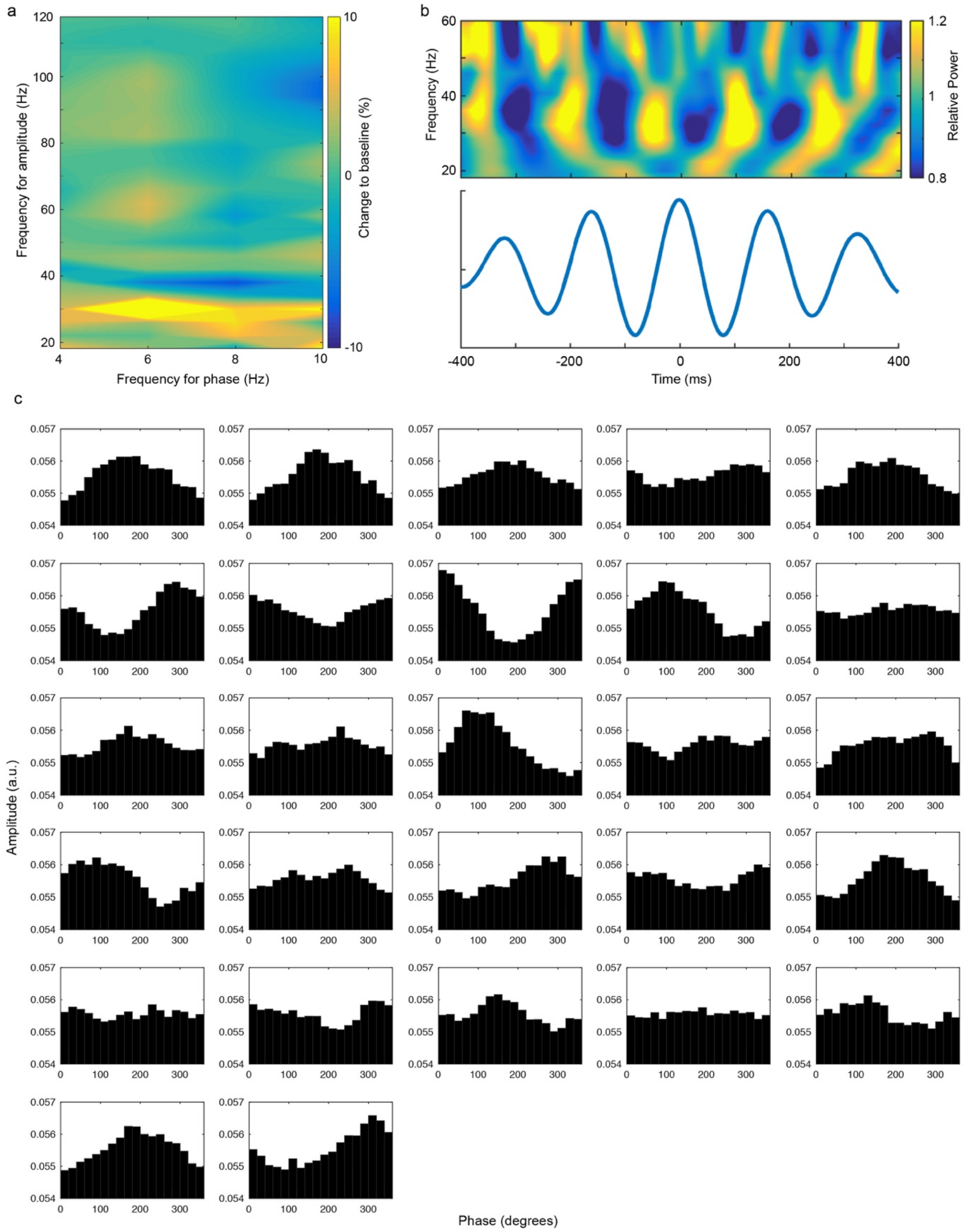

Figure S3. High-frequency power distributions over low-frequency phase. (a) Percent change of modulation indices during the averaged delay periods (condition average) as compared to the baseline window for all considered pairs of low and high frequencies in the cluster of sensors showing significant differences in PAC. (b) Time-resolved high-frequency power as a function of the underlying theta oscillation during the delay period from a single

participant. Power in the beta band depends on the phase of an underlying theta rhythm. (c) Beta amplitudes as a function of theta phase during the averaged delay periods within the cluster of sensors showing significant differences in PAC for all twenty-seven participants. The plots show that the beta power distribution over the theta phase was unimodal, and not multimodal, in all participants. They also illustrate that the individual power distribution varied across the theta phase in many participants. Such variations could arise from, for instance, different dipole orientations due to individual differences in cortical folding. Hence, the functional significance of this variability cannot be readily interpreted with MEG data. Nevertheless, we suppose that increased beta power indexes the "duty cycle"[2] of the theta rhythm, which might be represented by the peak or the trough of theta cycle depending on the orientation of the dipole in the cortex.

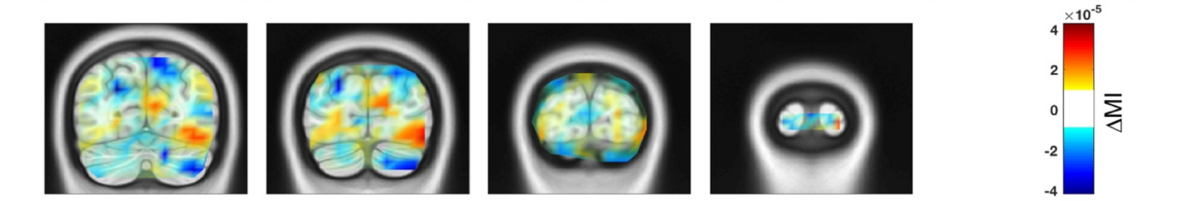

Figure S4. Unmasked whole-brain differences in theta-beta PAC between the averaged delay periods and the baseline window. Cluster-based permutation statistics revealed significantly enhanced theta-beta PAC in a cluster of voxels spanning from left MTL to left IFG (circled; also see Fig. 3c in the manuscript). No other cluster of voxels showed significant differences in PAC in any of the contrasts. MI: modulation index.

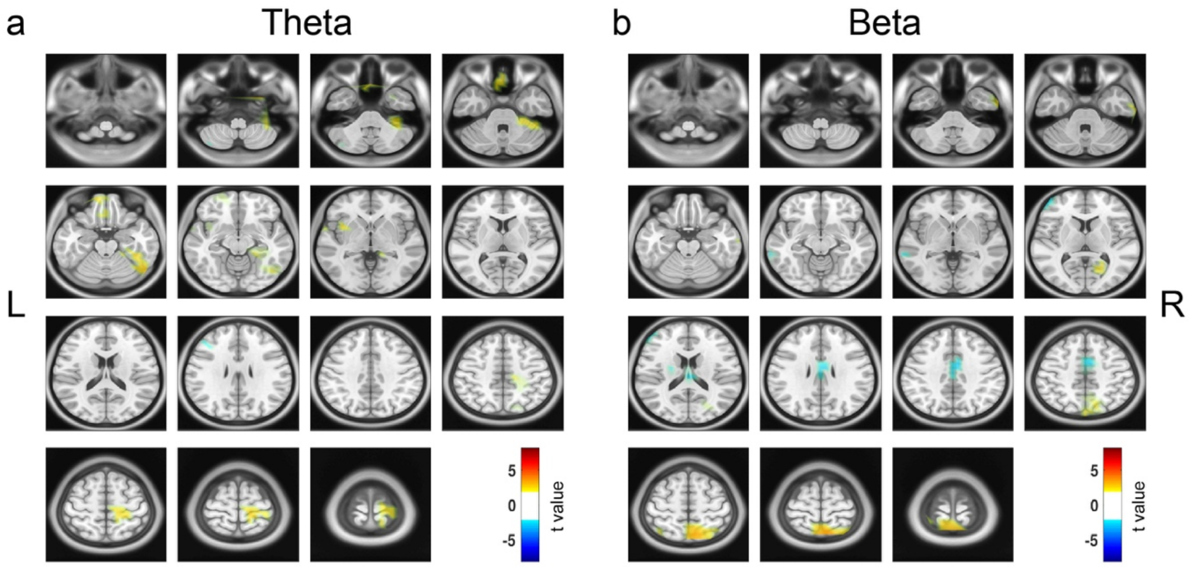

Figure S5. Uncorrected whole-brain power differences between the dual-task WM conditions (VA) and the single-task WM condition (V_{only}) for the two frequency bands showing differences in imaginary coherence. Whole-brain statistical power differences ($p < 0.05$, uncorrected) in the (a) theta band as well as the (b) beta band. There were no significant power differences in any of the two frequency bands that could explain the statistically significant differences in imaginary coherence from MTL to lPFC in the theta nor from MTL to temporooccipital regions in the beta band.

References

1. Daume, J., Gruber, T., Engel, A. K. & Friese, U. Phase-amplitude coupling and long-range phase synchronization reveal frontotemporal interactions during visual working memory. *J. Neurosci.* **37,** 313–322 (2017).

2. Jensen, O. & Mazaheri, A. Shaping functional architecture by oscillatory alpha activity: gating by inhibition. *Front. Hum. Neurosci.* **4,** 1–8 (2010).
